# Prevalence and clinical correlates of somatic mutation in aldosterone producing adenoma-Taiwanese population

**DOI:** 10.1038/srep11396

**Published:** 2015-06-12

**Authors:** Vin-Cent Wu, Kuo-How Huang, Kang-Yung Peng, Yao-Chou Tsai, Che-Hsiung Wu, Shuo-Meng Wang, Shao-Yu Yang, Lian-Yu Lin, Chin-Chen Chang, Yen-Hung Lin, Shuei-Liong Lin, Tzong-Shinn Chu, Kwan-Dun Wu

**Affiliations:** 1 Department of Internal Medicine, National Taiwan University Hospital and National Taiwan University College of Medicine, Taiwan; 2 Urology, National Taiwan University Hospital and National Taiwan University College of Medicine, Taiwan; 3 Division of Urology, Buddhist Tzu Chi General Hospital, Taipei Branch, Taiwan; 4 Internal Medicine, Buddhist Tzu Chi General Hospital, Taipei Branch, Taiwan; 5 Medical Imagine, National Taiwan University Hospital and National Taiwan University College of Medicine, Taiwan

## Abstract

Primary aldosteronism (PA) is a common form of secondary hypertension and has significant cardiovascular consequences. Mutated channelopathy due to the activation of calcium channels has been recently described in aldosterone-producing adenoma (APA). The study involved 148 consecutive PA patients, (66 males; aged 56.3 ± 12.3years) who received adrenalectomy, and were collected from the Taiwan PA investigator (TAIPAI) group. A high rate of somatic mutation in APA was found (n = 91, 61.5%); including mutations in *KCNJ5* (n = 88, 59.5%), *ATP1A1* (n = 2, 1.4%), and *ATP2B3* (n = 1, 0.7%); however, no mutations in *CACNA1D* were identified. Mutation-carriers were younger (<0.001), had lower Cyst C (p = 0.042), pulse wave velocity (p = 0.027), C-reactive protein (p = 0.042) and a lower rate of proteinuria (p = 0.031) than non-carriers. After multivariate adjustment, mutation carriers had lower serum CRP levels than non-carriers (p = 0.031. Patients with mutation also had a greater chance of recovery from hypertension after operation (p = 0.005). A high incidence of somatic mutations in APA was identified in the Taiwanese population. Mutation-carriers had lower CRP levels and a higher rate of cure of hypertension after adrenalectomy. This raises the possibility of using mutation screening as a tool in predicting long-term outcome after adrenalectomy.

Primary aldosteronism (PA), characterized by the autonomous production of aldosterone, is a common potentially curable cause of secondary hypertension^1^. It affects 5–13% of patients with hypertension in specialized centers^2^,^3^. PA patients have a high incidence of cardiovascular events in comparison with essential hypertension (EH) patients^4^,^5^. Somatic mutations in the selectivity filter of the KCNJ5 channel in APA result in a loss of selectivity for potassium and the entry of sodium, resulting in membrane depolarization, calcium mobilization, increased *CYP11B2* expression, and aldosteronism^6^. Further exome sequencing of aldosterone producing adenoma (APA) has identified ATPase mutations in *ATP1A1* and *ATP2B3,* which could lead to the opening of voltage-dependent calcium (Ca^2+^) channels^7^,^8^. The *CACNA1D* mutation induced a shift in voltage-dependent gating to a more negative voltage, again implicating gain-of-function Ca^2+^ channel mutations in APAs^9^,^10^. Therefore, aldosterone secretion is regulated by the augmentation of intracellular calcium levels via channelopathies and pumps, which lead to depolarization of zona glomerulosa cells in the adrenal cortex^11^,^12^.

Although aldosterone is now recognized as an increasingly important contributor to cardiometabolic pathology via informatory non-genomic pathways, in addition to its classically described genomic role in sodium and volume-related regulation^13^,^14^, its effect on cardio-metabolic factors in patients harboring these mutations has yet to be characterized. Previous findings are contradictory with regard to whether KCNJ5 mutations translate into a specific phenotype^15^. In this study, we have analyzed 148 APAs, diagnosed and surgically removed by the Taiwan Primary Aldosteronism investigator (TAIPAI) study group, for somatic mutations in *KCNJ5, ATP1A1, ATP2B3* and *CACNA1D*. We searched for clinical correlations and determined the impact on patients who harbored these mutations, focusing on their cardio-metabolic properties.

## Materials and methods

### Ethics Statement

The study complied with the Declaration of Helsinki and was approved by the institutional review board of National Taiwan University Hospital (Taipei, Taiwan) (No. 200611031R). All participants signed the informed consent form before inclusion in the study. All of the methods were carried out in “accordance” with the approved guidelines ( http://www.nature.com/srep/policies/index.html#experimental-subjects).

### Identification of PA

The present study was based on the Taiwan Primary Aldosteronism Investigation (TAIPAI) database and tissue bank^16^,^17^,^18^. The database was constructed from June 2008 to March 2011 for quality assurance at 2 medical centers and their 3 affiliated hospitals and two local hospitals in different cities in Taiwan^19^. Before confirmatory tests, all antihypertensive medications were discontinued for at least 21 days. Diltiazem and/or doxazosin were administered to control markedly high blood pressure when required^1^. Patients with an abnormal aldosterone-renin ratio (ARR) were confirmed with PA by saline infusion tests, and subsequently underwent imaging studies for subtype identification (figures S1 and S2).

PA confirmation and subtype studies were established in hypertensive patients according to the standard protocol of TAIPAI^59^, including adrenal venous sampling and NP-59 scintigraphy with SPECT-CT imaging^16^,^17^,^18^,^20^ (see the supplementary file). Patients who were diagnosed with family type I (FH-I)/GRA were excluded via long-range polymerase chain reaction, as described previously^21^.

### Functional survey

The aldosterone concentration was measured by radioimmunoassay using a commercial kit (Aldosterone Maia Kit, Adaltis Italia S.P.A., Bologna, Italy)^22^ and PRA was measured by the generation of angiotensin I *in vitro* using a commercially available radioimmunoassay kit (DiaSorin, Stillwater, MN, USA)^19^. The amount of daily protein loss was defined as the urinary microalbumin-to-creatinine ratio (mg/mg); the cardiovascular marker cystatin C^11^ was measured using a particle-enhanced immunonephelometric assay (N Latex Cystatin C; Siemens, Berlin, Germany) with a nephelometer (BNII; Siemens)^23^. The pulse wave velocity (PWV) was measured with the subject in a supine position after a 15-min rest using an automatic waveform analyzer (Colin VP-2000, Omeron Inc., Japan), as previously reported^24^.

A Hewlett-Packard Sonos 5500 ultrasound system equipped with an S3 transducer was used for Echocardiography, including two-dimensional, M-mode and Doppler ultrasound recordings. Left atrial diameter and left ventricular ejection fraction (M-mode) were measured via the parasternal long-axis view, as in our previous report^25^. Left ventricular mass (LVM) index (LVMI) was calculated according to the method of Devereux *et al.*^26^.

### Adrenalectomy

The indication for adrenalectomy is according to AVS (n = 65) or NP 59 (n = 88) lateralization (supplementary file). All of the operations were performed using the lateral trans-peritoneal approach, and were performed by a single experienced laparoscopic surgeon to ensure that the principles of adrenal gland surgery were strictly followed^27^.

### Sequencing

#### Nucleic acid extraction

The adrenal tumors obtained were fresh-frozen and stored at −72 ^o^C. Genomic DNA was extracted from 148 paired peritumoral adrenal cortices in addition to each peripheral DNA sample. Tumor DNA was extracted using a QIAamp DNA mini kit (Qiagen, Hilden, Germany); total RNA was isolated from frozen tissue using Trizol (Invitrogen, Carlsbad, Ca, USA) and then cleaned-up using the GENEzol TriRNA Pure Kit (Geneaid, New Taipei City, Taiwan). After DNaseI treatment (Invitrogen, Carlsbad, CA, USA), 500 ng of total RNA were reverse-transcribed using Moloney Murine Leukemia Virus Reverse Transcriptase (M-MLV RT) (Promega, Madison, WI, USA) and random hexamers (Promega, Madison, WI, USA) according to the manufacturer’s instructions.

#### Somatic mutation sequencing

The coding region of the genomic DNA was investigated by exome sequencing. The entire coding sequence (exons 2–3) and flanking regions of *KCNJ5* were amplified and sequenced using gene-specific primers, as previously reported^28^. Accordingly, the PCR primers used to amplify fragments for direct sequencing of *ATP1A1/ATP2B3* and *CACNAD1* also followed previous reports^7^,^9^,^29^ (listed in table S1). The annealing temperature was 58 °C. Direct sequencing of PCR products was performed using The BigDye® Terminator v3.1 Cycle Sequencing Kit (Applied Biosystems, Foster City, USA) with a 3730 DNA Analyzer (Applied Biosystems, Foster City, USA).

#### Outcome measurement

Patients were followed monthly for the first three months after the operation, and then every three months. Assessment of cure rate of hypertension has been described previously^30^. Briefly, hypertension was considered cured if 75% of their recorded systolic BP was <140 mmHg and their diastolic BP was <90 mmHg with no use of anti-hypertensive medications for at least 1 year after adrenalectomy^30^,^31^.

### Statistical analysis

Statistical analyses were performed with R software, version 2.8.1 (Free Software Foundation, Inc., Boston, MA, U.S.A.). A normal distribution was attained by the appropriate transformations of skewed variables such as C-reactive protein (CRP) and ARR. Logistic regression analysis with a **stepwise** variable selection procedure was applied using available variables (body mass index (BMI), diabetes mellitus, preoperative aldosterone, potassium blood pressure, kidney function, CRP, proteinuria, somatic mutation result) to identify important factors associated with postoperative residual hypertension. The goodness-of-fit (GOF) of the fitted multiple logistic regression model was assessed by the estimated area under the receiver operating characteristic (ROC) curve, the adjusted generalized *R*^2^, and the Hosmer-Lemeshow GOF test. Furthermore, LOESS curves^32^ were drawn to evaluate the relationship between age with CRP and PWV.

Because of dis-equivalent ages and CRP between groups and to display the implications of variables for individual patients, a generalized additive model (GAM) (with spline) incorporating the subject-specific (longitudinal) random effects was plotted, adjusted for the factors listed in Table 1. We used a transformation of the β coefficients [100* (e^β^ −1)] to obtain the percentage increase in the outcomes per SD increase in variables. This approach permits adjustments for possible nonlinear effects of continuous variables^24^,^33^.

All data were expressed as the mean ± standard deviation (SD). A p-value of <0.05 was considered significant.

## Results

### Patient characteristics

#### Demographics of Patients (Table 1)

Of the 148 patients (66 males; aged 56.3 ± 12.3years) identified who received adrenalectomy, a high rate of somatic mutation was found (n = 91, 61.5%), most of which were *KCNJ5* mutations (n = 88, 59.5%).

Sequencing of adenoma samples demonstrated the occurrence of the p.Gly151Arg (c.451G > A or c.451G > C) (n = 45), the p.Leu168Arg (c.503T > G)(n = 41), p.Ile157del (c.470_472delTCA) (n = 1), and p.Thr158Ala(c.472A > G) (n = 1) mutations in the heterozygous state. Two adenomas had nucleotide substitutions in *ATP1A1* (p.Leu104Arg [c.311 T > G]) and one in *ATP2B3* (p.Tyr410Asp [c. 1228 T > G]) (supplementary table 2).

There was no *CACNA1D* mutation found in our specimens. The absence of *KCNJ5* mutations in all peripheral DNA samples and paired peritumoral cortices confirmed the somatic nature of the genetic alteration.

Mutation carriers were younger (p < 0.001) compared with their mutation-free counterparts. The basal levels of serum PAC and the aldosterone to renin ratio (ARR) were higher, while potassium was lower in mutation-carriers than in non-carriers (all p < 0.001). Patients with these mutations were more likely to demonstrate a higher glomerular filtration rate (p = 0.001), estimated by the CKD-Epi formula, than mutation-free patients.

### Cardiometabolic parameters

**Figure 1:**
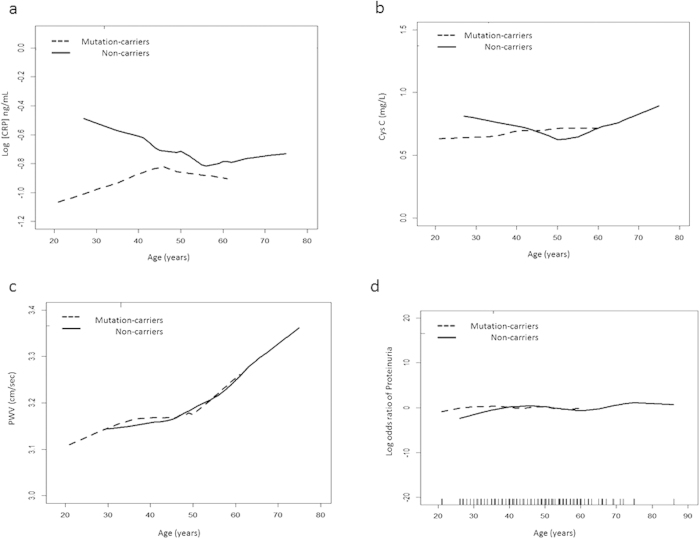
Scatter plots with an adjusted spline of age with (**a**) C reactive protein (CPR), ( p = 0.031 between mutation-carriers and non-carriers after multivariate adjustments) (**b**) Cystatin C (Cys C) and (**c**) pulse wave velocity (PWV). The plots were incorporated with the subject-specific (longitudinal) random effects to predict the association. The probability of proteinuria was constructed with chronological age (d) and is centered to have an average of zero over the range of the data as constructed with the generalized additive model (GAM). *Adjusted by sex, diabetes mellitus, body mass index (BMI), serum aldosterone, potassium, estimated glomerular filtration rate, mean blood pressure. **All plots were constructed and stratified by patients with somatic mutation and non-mutation.

Cyst C (p = 0.042), PWV (p = 0.027), CRP (p = 0.042) and proteinuria (p = 0.031) were lower in mutation-carriers than in the non-carriers group. However, the preoperative LV mass and LVEF were not significantly different between the groups. To compare these vascular parameters further between the mutation-carriers and non-carriers, we generated Loess plots stratified by chronological age with objects. In terms of the relationship of CRP to each age, the mutation-carriers had a lower CRP than the non-carriers after multivariate correlation (p = 0.031, Fig. 1). In the almost linear relationship between age and PWV, there was a steep increase in PWV up to 50 years in both groups. The relationship between PWV or Cystatin C and age showed no difference between the mutation-carriers and non-carriers after multivariate adjustments (all p > 0.05).

The probability of proteinuria associated with the chronological age of the aldosteronism patients at diagnosis was constructed with the GAM. The prevalence of proteinuria was also not different between the two groups after adjustment. Age did not relate to having proteinuria in either group (all p > 0.05).

### Factors predicting post-operative residual hypertension

After unilateral adrenalectomy, hypertension was cured in 125 (84.5%) patients. Most (n = 100, 80%) of the cured patients became normotensive within 6 months after surgery; twenty patients (16%) became normotensive after 9 months, and 5 took up to 1 year. Of the 23 patients with residual hypertension, 5 continued using the same antihypertensive agents.

**Figure 2:**
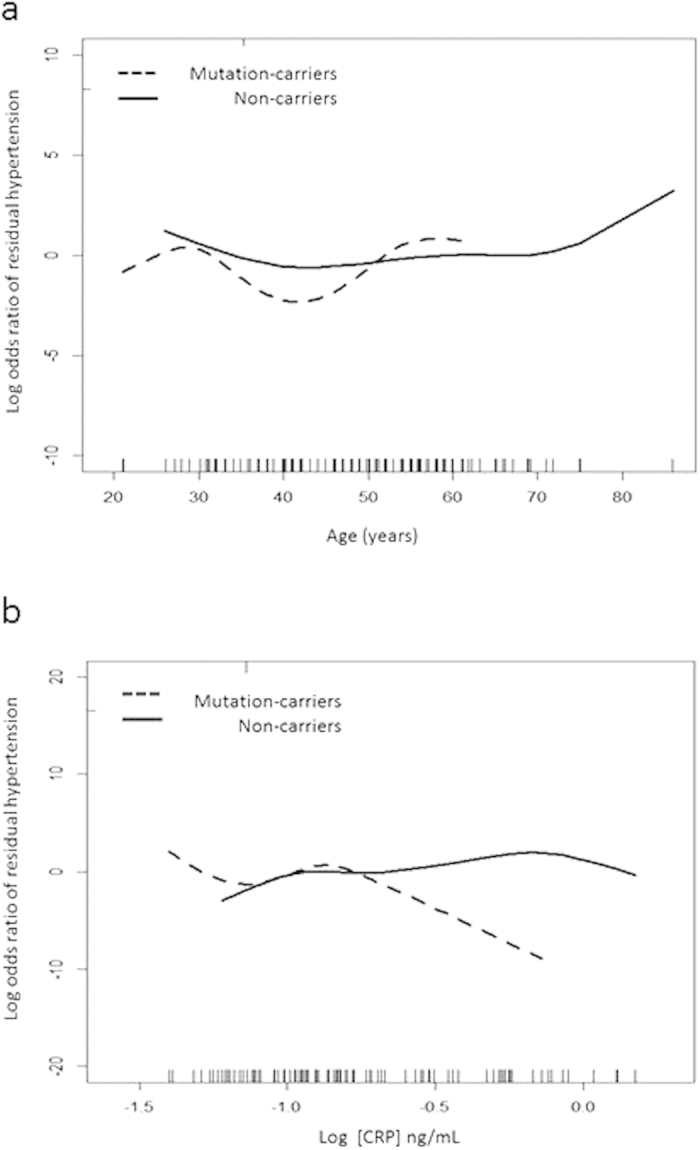
Generalized additive model (GAM) with the spline of (**a**) chronological age at diagnosis and (**b**) baseline c-reactive protein (CRP) level, incorporating the subject-specific (longitudinal) random effects, were plotted to predict the residual hypertension after adrenalectomy. The curve was centered to have an average of zero over the range of the data. *Adjusted by sex, diabetes mellitus, body mass index (BMI), serum aldosterone, potassium, estimated glomerular filtration rate, mean blood pressure. Figures 2a, the GAM figures of correlation with age and residual hypertension after adrenalectomy. Figures 2b, the GAM figures of correlation with CRP and outcome effects. (The response scale reflects natural transformation).

Compared with wild type individuals, the mutation-carriers had a greater chance of recovery from hypertension (92.3% vs 71.9%, p = 0.002) after a one year follow-up. Adjusted for possible variables, being a mutation-carrier [OR, 0.08, 95% CI, 0.01– 0.47, p = 0.005] was an independent factor predicting postoperative cure of hypertension (Table 2). However, preoperative logCRP [OR, 8.12, 95% CI, 1.12– 58.99, p = 0.039] and proteinuria [OR, 8.12, 95% CI, 1.02– 24.34, p = 0.048] were risks predicting postoperative residual hypertension. The final multiple logistic regression model had a high discriminatory power (estimated area under the curve of receiver operating characteristics [eAUC-ROC] = 0.807) and fitted the observed binary data well (adjusted generalized R^2^ = 0.349 and Hosmer-Lemeshow GOF test p = 0.074). The GAM curve was further plotted to show the differentiation of residual hypertension between mutation-carriers and non-carriers. Stratified by chronological age at diagnosis, non-carriers had a greater probability of residual hypertension than patients with a mutation after adrenalectomy in the model (Fig. 2a). This result was consistent in patients stratified by baseline CRP level; patients without a mutation had a higher probability of residual hypertension (Fig. 2b).

## Discussion

The main finding of this multicenter study is that nearly two thirds of these Taiwanese APA patients had candidate somatic mutations, almost all in *KCNJ5*, and the prevalence was higher than previously reported in Caucasian subjects^29^,^34^. There were no gender differences in mutation carriers or non-carriers. Our results reinforce the view that patients with somatic mutations are younger; however, mutation carriers and non-carriers had similar degrees of arterial stiffness, proteinuria and left ventricular hypertrophy after multivariate adjustment, suggesting that, despite mutation carriers having an earlier onset of the disease, this does not translate into a unique cardiovascular phenotype^35^,^36^. Most importantly, we demonstrated that those patients with somatic mutation have a lower level of pro-inflammatory status independent of chronological age, and are more likely to be cured post-operatively.

### Mutation Carriers

The prevalence of being a mutation carrier was 61.5% among adenomas, most of which were in *KCNJ5* (59.5%); this is a higher rate than previously reported^29^,^36^. We showed that *KCNJ5* mutations are the most frequent genetic cause of excessive aldosterone production in Eastern Asian patients with APA. Heterozygous mutations in the conservative filter of *KCNJ5* at p.Gly151Arg were the most common channelopathy in our cohort. There was no mutated *CACNA1D* identified. To date, the three categories of mutation in adenoma have been mutually exclusive. Likewise, nearly three-fifths of our patients had a KCNJ5 mutation, meaning that it was less likely that there would be other mutations. In contrast, the use of AVS in a relative minority of patients with PA (43.9%) could have been responsible for the extremely low prevalence of CACNA1D mutations in our cohort. In fact, smaller nodular dimensions were found in patients harboring CACNA1D mutations^10^.

Indeed, both the current and previous studies have shown that mutation carriers had higher preoperative plasma aldosterone levels and lower potassium compared with patients without mutation^35^,^36^,^37^. This may explain why mutation carriers were likely to be diagnosed at a younger age. However, our findings could not evaluate the possibility that the etiology of PA diagnosed at a younger age may be attributed to mutations. We found no significant differences in preoperative blood pressure, body mass or tumor size between the two groups. This suggests that harboring a mutation brings the presentation of aldosteronism forward and may be indicative of a more florid phenotype.

However, non-somatic mutation carriers had higher C-reactive protein levels than mutation carriers. Although recently identified mutations in the Kir3.4 (*GIRK4*) gene coding for KCNJ5 channels were shown to represent a key mechanism for the aldosterone excess in part of APA patients, the mechanism underlying the aldosterone overproduction in non-mutation carriers is still not clear. The overproduction of oxidative stress in aldosteronism^38^,^39^ is not only attributed to cardiovascular injury but could have an impact on redox-activation of G-protein-activated inward rectifier K^+^ channels^40^ in non-mutation carriers, at least partially as a key pathophysiology in oxidative stress-associated aldosterone secretion. It was found that G-protein-activated, inwardly rectifying K^+^ channel subunit GIRK is a potential target for oxidative stress^40^. To date, a variety of membrane-bound proteins like Na^+^/K^+^-ATPase or adenylate cyclase are known to be affected by reactive oxygen species (ROS) and are probably involved in noxious Ca^2+^ overload^41^,^42^. In particular, K^+^-selective channels are prone to operational modulation by ROS, and redox regulation of K^+^ channels as a mechanism that provides a link between cellular metabolism and excitability^43^,^44^. The mechanism of high CRP in non-mutation carriers and its impact on channelopathy is still not clear; therefore, the hypothesis that oxidative stress is a causative factor for the generation of autonomous aldosterone production still needs further study.

### *Factors related to residual hypertension after adrenalectomy*

Importantly, we found that mutation-carriers had better odds of recovery from hypertension after adrenalectomy, a finding which is in concordance with two recent smaller studies from Western Norway^45^ and a center in Australia^46^. Patients with *KCNJ5* mutations were reported to have a higher likelihood of achieving regression of LVH^35^. Our results showed that among younger patients receiving adrenalectomy, mutation carriers were more likely to be cured of hypertension, while non-carriers often had residual hypertension, despite adjusting for age (Fig. 2a. GAM plot). This result was especially true for younger ages, and was therefore not only age-related essential hypertension. We have further shown that patients who have proteinuria will have a lower chance of recovery from hypertension after adrenalectomy. Heavy proteinuria in PA patients, which is associated with less renin suppression^47^, showed a lower possibility of recovery from kidney damage after adrenalectomy^47^.

### Cardiometabolic parameters

Patients harboring mutations had lower cystatin C, PWV and CRP levels and a lower percentage of proteinuria than non-carriers. However, there were no differences in cystatin C, proteinuria, and PWV between groups after adjustments in our final model. These findings contradict the contention that PA mutations do not translate into a specific phenotype^36^. However, in the multivariate model, non-carriers had higher CRP than mutation carriers and CRP could predict post-operative residual hypertension. It has been noted that high pro-inflammatory status is related to vascular hypertrophy^48^,^49^, endothelial dysfunction^50^ and cardio-metabolic syndrome^51^.

Several mechanisms have been proposed to explain residual hypertension after adrenalectomy, such as endothelial dysfunction, vascular damage and arteriolosclerosis^52^,^53^. In a European report, non-carriers in the APA were less likely to achieve regression of left ventricle hypertrophy^35^, partially reflecting their long-term exposure to a higher pro-inflammation status. It has been shown that aldosterone promotes oxidative stress through a non-genomic effect and is mineralocorticoid receptor (MR)-independent^54^, i.e. cannot be attenuated by the classic MR-blocker spironolactone. Importantly, the effect of mutated channelopathy and pumps on CRP levels persisted after adjustments for level of PAC and potassium level. Thus, it appears that the possibility of identifying APA patients who are at risk of developing a pro-inflammatory status early is attractive because the cardiovascular risks could be reversed and hypertension cured by further targeted management in the early stages of disease. Future studies should determine the long-term cardiometabolic events in patients with or without mutations, especially the effect of non-genomic actions.

### Higher prevalence of mutation-carriers

In contrast to previous studies conducted in other ethnic groups, mutations were detected in a relatively higher percentage of Taiwanese patients; also, the patterns of mutations were shown to be different. The largest cohort from Asia in this study and also that in Japanese reports^37^,^55^,^56^ with a higher prevalence of mutation-carriers showed no gender differences, which is different to Caucasian reports^6^,^29^,^36^. Although a plausible explanation for the difference in the phenotype between sexes has been reported to be the androgen-regulated compensatory expression of TASK3 channels, our results did not show a sex difference phenotype in Eastern Asian patients. This suggests the presence of epidemiological differences between Asian and Western populations. This high frequency of *KCNJ5* mutations may partially contribute to the increased prevalence of adenoma compared with hyperplasia in Asian individuals^17^,^55^,^57^. The variations in phenotypes resulted from different mutations in *KCNJ5* also have implications for the potential channelopathy of PA^15^. Under the standard diagnostic implementation criteria and possessing the same ethnic background, the patients enrolled and sample collections in this study were the same between the centers involved. Using standard methods, with the higher percentage of APA identified^17^, the higher rate of *KCNJ5* mutations in our adenomas is less likely to be related to differences in the approach and treatment methods^17^.

Although unilateral adrenalectomy represents the treatment of choice for lateralized PA, it is tempting to speculate that channelopathy and pumps may become interesting new drug targets for some subgroups of APAs that are not eligible for surgery, e.g. blocked by verapamil^58^, especially in areas with a higher prevalence of mutation-carriers.

There are some limitations of this study. As most of the mutations were in *KCNJ5,* with a small number of patients with ATPase mutations, but no *CACNA1D* mutations in our samples, analysis of clinical and biological correlates of these rare mutations could not be performed. The role of sex hormones such as estrogen and progesterone on channelopathy and pumps in PA patients warrants further study. Prospective studies evaluating the predictive value of mutation screening for outcomes in patients undergoing adrenalectomy for PA will also be necessary.

## Conclusions

We sought to provide a genotype-phenotype perspective in correlating mutated status with clinical outcome and cardio-metabolic parameters used in APA patients from an area with high mutation rates. Lower CRP levels in mutation-carriers relates to a higher rate of cure of hypertension after adrenalectomy. It also raises the possibility of using mutation screening as tool in determining long-term outcome after surgery.

## Additional Information

**How to cite this article**: Wu, V.-C. *et al.* Prevalence and clinical correlates of somatic mutation in aldosterone producing adenoma -Taiwanese population. *Sci. Rep.* **5**, 11396; doi: 10.1038/srep11396 (2015).

## Supplementary Material

Supplementary Information

## Figures and Tables

**Table 1:**
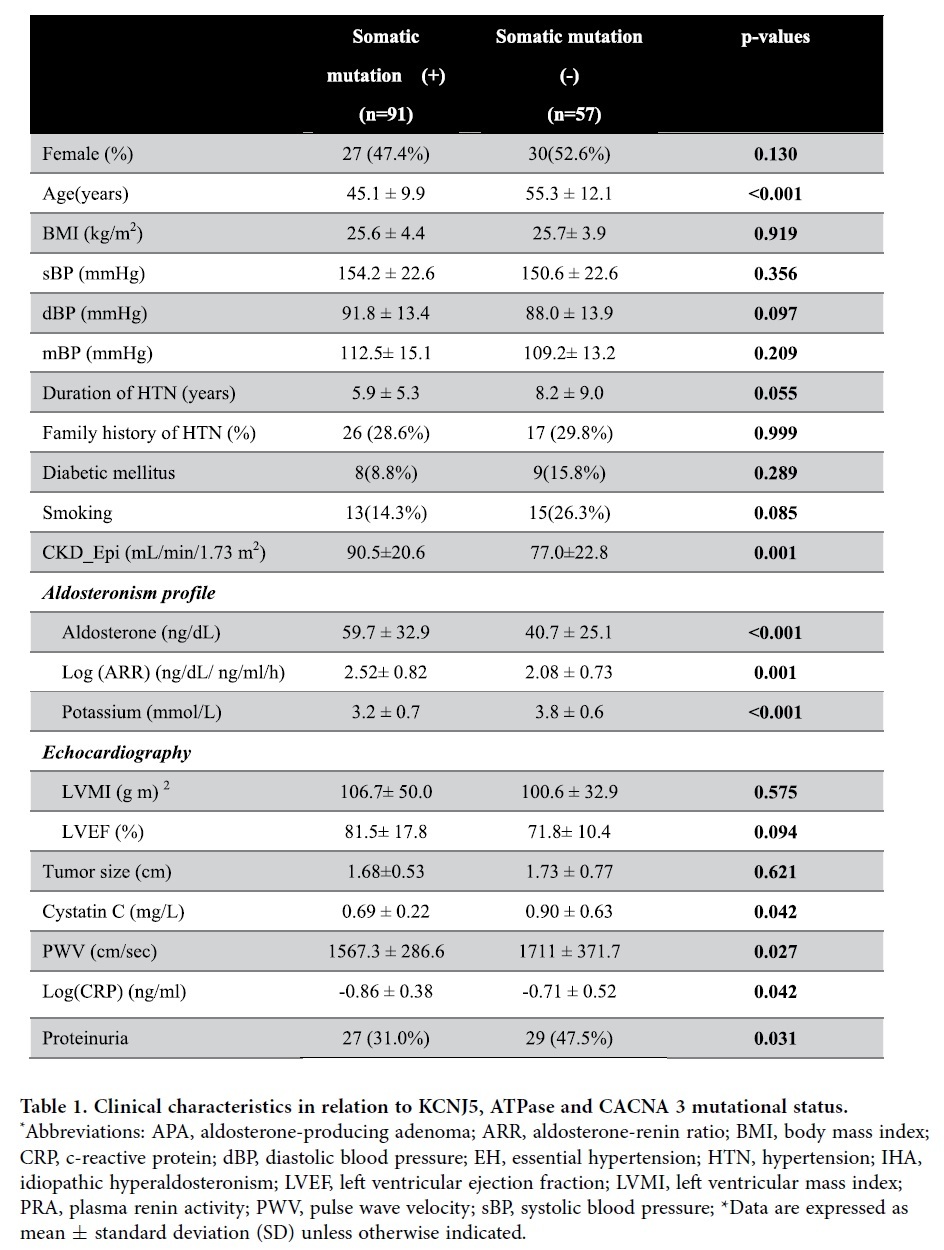
Clinical characteristics in relation to KCNJ5, ATPase and CACNA 3 mutational status. ^*^Abbreviations: APA, aldosterone-producing adenoma; ARR, aldosterone-renin ratio; BMI, body mass index; CRP, c-reactive protein; dBP, diastolic blood pressure; EH, essential hypertension; HTN, hypertension; IHA, idiopathic hyperaldosteronism; LVEF, left ventricular ejection fraction; LVMI, left ventricular mass index; PRA, plasma renin activity; PWV, pulse wave velocity; sBP, systolic blood pressure; *Data are expressed as mean ± standard deviation (SD) unless otherwise indicated.

**Table 2:**
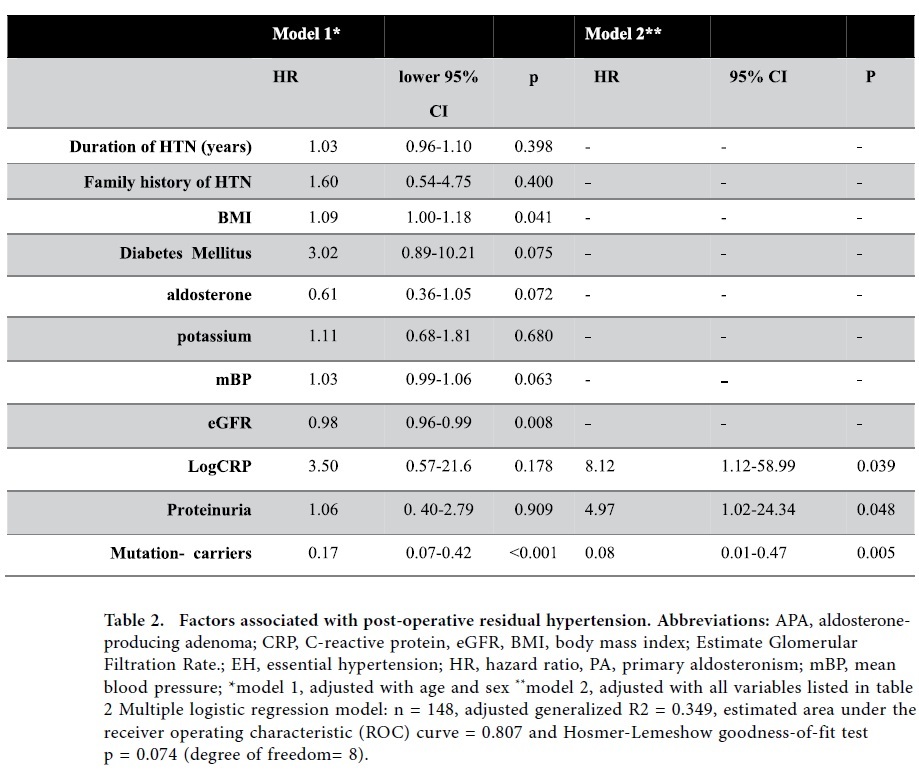
Factors associated with post-operative residual hypertension. Abbreviations: APA, aldosterone-producing adenoma; CRP, C-reactive protein, eGFR, BMI, body mass index; Estimate Glomerular Filtration Rate.; EH, essential hypertension; HR, hazard ratio, PA, primary aldosteronism; mBP, mean blood pressure; ^*^model 1, adjusted with age and sex ^**^model 2, adjusted with all variables listed in table 2 Multiple logistic regression model: n = 148, adjusted generalized R2 = 0.349, estimated area under the receiver operating characteristic (ROC) curve = 0.807 and Hosmer-Lemeshow goodness-of-fit testp = 0.074 (degree of freedom= 8).
