# Supplementary material for: Prevalence and clinical correlates of somatic mutation in aldosterone producing adenoma-Taiwanese population
Source: Sci Rep. 2015 Jun 12;5:11396. doi: 10.1038/srep11396 (PMC4464349; doi:10.1038/srep11396)
Supplement: Supplementary Information [file srep11396-s1.doc]

**Prevalence and clinical correlates of somatic mutation in aldosterone producing adenoma****-Taiwanese population**

Vin-Cent Wu^1^, Kuo-How Huang^2^, Kang-Yung Peng^1^, Yao-Chou Tsai^3^, Che-Hsiung Wu^4^, Shuo-Meng Wang^2^, Shao-Yu Yang^1^, Lian-Yu Lin^1^, Chin-Chen Chang^5^, Yen-Hung Lin^1^, Shuei-Liong Lin^1^, Tzong-Shinn Chu^1^, and Kwan-Dun Wu^1^

**Supplementary data**

***Material and methods***

**Enrolled hospitals**

This study included two medical centers (National Taiwan University Hospital (NTUH), Taipei; Taipei University Hospital , Taipei), three metropolitan hospitals (Cardinal Tien Hospital, New Taipei City; Taipei Tzu Chi Hospital, New Taipei City; Yun- Lin Branch of NTUH, Douliou City), and two local hospitals (Hsin-Chu Branch of NTUH, Hsin-Chu City; Zhongxing Branch of Taipei City Hospital, Taipei).

**The standard protocol to identify aldosteronism**

The diagnosis of aldosteronism was established in hypertensive patients on the basis of the following criteria: (Fig S1)

***Screen***

We performed PA test in with the following features: 1) Joint National Commission stage 2 (systolic/diastolic more than 160 – 179/100 – 109 mmHg) and stage 3 (more than 180/110 mmHg) hypertension; 2) drug-resistant hypertension; 3) hypertension with spontaneous hypokalemia or diuretic-induced hypokalemia; 4) hypertension with adrenal incidentaloma; 5) hypertension and a family history of early-onset hypertension, or cerebrovascular accident at a young age ( more than 40-year-old); and 6) patients with first degree relatives diagnosed with PA.

***Confirmation***

Fulfillment of the following three conditions confirms a diagnosis of aldosteronism: (1) autonomous excess aldosterone production evidenced with an ARR > 35; (2) a TAIPAI score larger than 60%; (3) post-saline loading PAC > 10 ng/dl. (Abbreviations: PAC, plasma aldosterone concentration; PRA, plasma renin activity)

***Lateralization***

1. APA is identified on the basis on the following four conditions: (1) autonomous excess aldosterone production evidenced with an ARR > 35, a TAIPAI score larger than 60%, and post-saline loading PAC > 10 ng/dl; (2) adenoma evidenced with a CT scan for pre-operative evaluation;[6] (3) lateralization of aldosterone secretion at AVS or during dexamethasone suppression NP-59 SPECT/CT; (4) pathologically proven adenoma after an adrenalectomy for those with operations, and subsequent emergence of either a cure pattern of hypertension without anti-hypertensive agents or improvement in hypertension, potassium, PAC, and PRA.
2. Idiopathic hyperaldosteronism (IHA) is distinguished on the basis on the following four criteria: (1) autonomous excess aldosterone production evidenced with an ARR > 35, a TAIPAI score larger than 60%, and post-saline loading PAC > 10 ng/dl (2) evidence of bilateral diffuse enlargement indicated by a CT scan for pre-operative evaluation; (3) non-lateralization of aldosterone secretion at AVS or during dexamethasone suppression NP-59 SPECT/CT; (4) evidence of diffuse cell hyperplasia reported in following pathology studies for those with operations.

**Figure S1**. The subtype-differentiating protocol of the TAIPAI group .


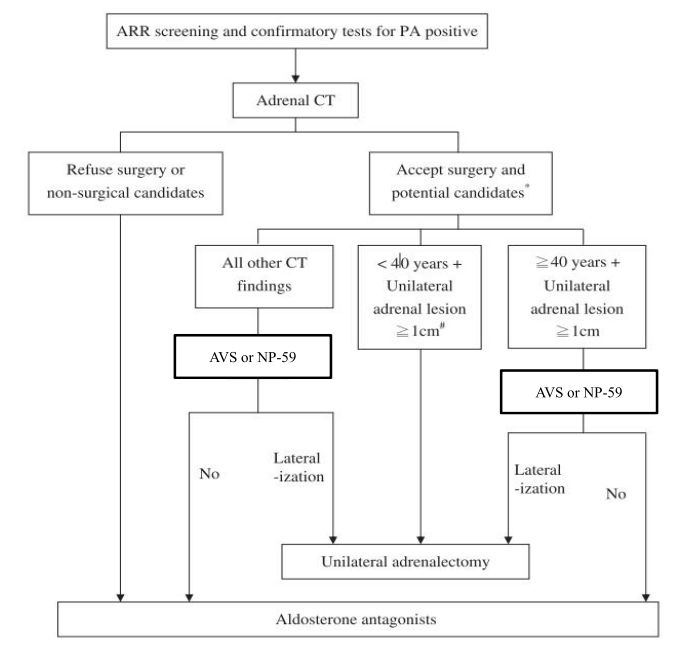


* Abbreviations: AVS, adrenal venous sampling; APA, aldosterone-producing adenomas; IHA, idiopathic hyperaldosteronism; NP-59 (SPECT/CT), I131-6b-iodomethyl-19-norcholesterol / SPECT/CT; INC, incidentaloma.

Operation

The indication for adrenalectomy is according to AVS or NP 59 lateralization.

A successful venous cannulation was noted if the ratio of the cortisol level of the adrenal vein to the inferior vein cava more than 3. Lateralization of aldosterone secretion was defined as a difference in the aldosterone/cortisol ratio greater than fourfold between the bilateral adrenal glands as our previously report .

**Figure S2**. Flow diagram of selecting study subjects.


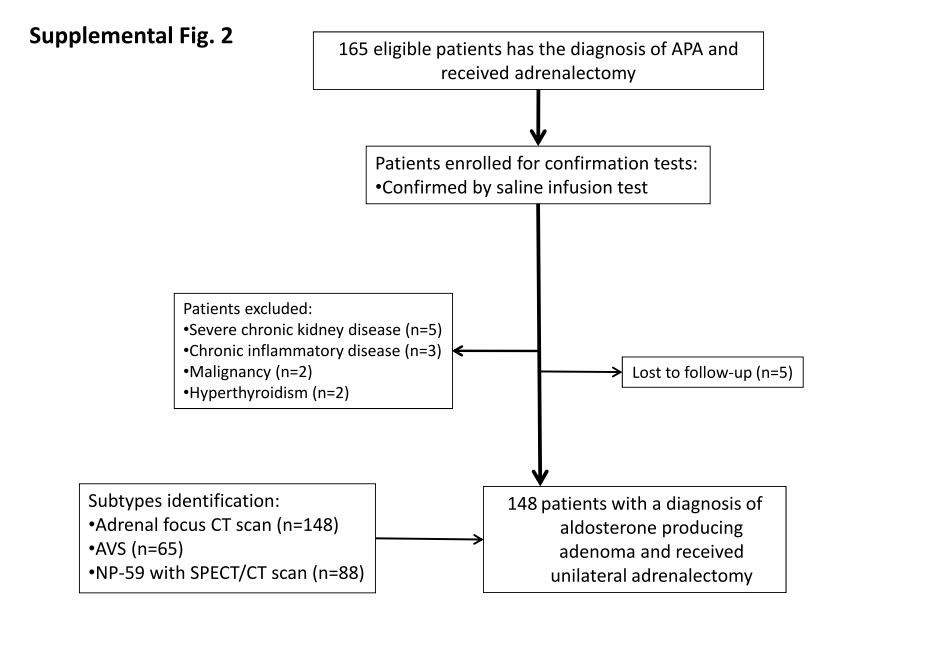


Membership of the Taiwan Primary Aldosteronism Investigation (TAIPAI) Study Group: Che-Hsiung Wu, MD(Chi-Taz, PI of Committee); Vin-Cent Wu, MD(NTUH, PI of Committee); Yen-Hung Lin, MD(NTUH, PI of Committee); Yi-Luwn Ho, MD, PhD(NTUH, PI of Committee); Hung-Wei Chang, MD, PhD(Far eastern hospital, PI of Committee); Lian-Yu Lin MD, PhD(NTUH, PI of Committee); Fu-Chang Hu, MS, ScD,Harvard statitics, Site Investigator); Kao-Lang Liu, MD(NTUH, PI of Committee); Shuo-Meng Wang, MD(NTUH, PI of Committee); Kuo-How Huang, MD(NTUH, PI of Committee); Yung-Ming Chen, MD(Yun-Lin Branch, NTUH, PI of Committee); Chin-Chi Kuo; MD(Yun-Lin, PI of Committee), Chin-Chen Chang, MD(NTUH, PI of Committee); Shih-Chieh Chueh, MD, PhD(Cleveland Clinic, , PI of Committee); Shih-Cheng Liao, MD(NTUH, PI of Committee); Ruoh-Fang Yen, MD, PhD(NTUH, PI of Committee); and Kwan-Dun Wu, MD, PhD(NTUH, Director of Coordinating Center).

Supplementary Table S1.

Primer sequences

| **Primer name** | **Primer sequence (5’→3’)** |
| --- | --- |
| KCNJ5_1F | GATGGTGTCTTTTTAACTCAAAGC |
| KCNJ5_1R | GTGATGACTCGGAAGCCATACC |
| KCNJ5_2F | CTTTCCTGTTCTCCATTGAGACC |
| KCNJ5_2R | CTGAGGAGGACAAAGCGCC |
| KCNJ5_3F | ATGCATGTAACTTCCGTTTCCC |
| KCNJ5_3R | GCCAGTGACAGGAGGTCTTAGG |
| KCNJ5_4F | CTTCATTTGGTGGCTCATTGC |
| KCNJ5_4R | GGGACTTGATGAGCTTGGC |
| ATP1A1_4F | TTCCTTGGGCCTATTGTTTG |
| ATP1A1_4F | GTGGGAGACAAAGACGGAGA |
| ATP1A1_8F | CGTGGCTTCCTTCAGGTTAG |
| ATP1A1_8R | CGTGATGTGGCTCTCAAGAA |
| ATP2B3_8F | CCTGGGCTGTTTATCCTGAA |
| ATP2B3_8R | CCCCA GTTTC CGAGT CTGTA |
| CACNA1D_8aF | AGCTGCAACTGGGGCTC |
| CACNA1D_8aR | GCAGCTAGGAGACACGCAG |
| CACNA1D_17F | TTTACTTCTGTAGACTGTCCTTTTA |
| CACNA1D_17F | ACACGTGACTCCCACTCTCAGC |
| CACNA1D_6F | GTAAAGGAGGCATGGTTAGG |
| CACNA1D_6R | TGGCTCAGTAAATGTGCTGGT |
| CACNA1D_8bF | GCCTTGATGACTCTGTGTG |
| CACNA1D_8bR | CCAGCAAAGCTTGTGTGGT |
| CACNA1D_23F | CACGCTAACTGTGCAGGGA |
| CACNA1D_23R | TCAGCTCTGCCCAGAAGAG |
| CACNA1D_27F | CCAATCTACAACCACCGCGT |
| CACNA1D_27R | GACCAAGGGACAGAAGCCAA |
| CACNA1D_32F | ACGGTTCTTCCTCACTGTCG |
| CACNA1D_32R | CTTCAGCAGAGGCATTTGGCT |

Supplementary Table S2. The clinical characteristics of ATPase mutations

| Pt ID | mutation | Sex | Age | APA diameter (mm) | AVS | NP59 | Number of nodule | Aldosterone  (ng/dl) | Potassium  mmole/dl | Categories before OP |
| --- | --- | --- | --- | --- | --- | --- | --- | --- | --- | --- |
| Pt1 | ATP1A1 | M | 72 | 15 | lateralization | - | 1 | 85.5 | 3.2 | 3 |
| Pt2 | ATP1A1 | M | 58 | 11 | lateralization | - | 1 | 114 | 3.4 | 3 |
| Pt3 | ATP2B3 | F | 29 | 11 | lateralization | lateralization | 1 | 68.5 | 3.7 | 1 |

**Abbreviation**

AVS, adrenal venous samplin, NP59, I131-6b-iodomethyl-19-norcholesterol / SPECT/CT, OP, operation, Pt, patients

**Referance**

1. Sechi LA, Novello M, Lapenna R, Baroselli S, Nadalini E, et al. (2006) Long-term renal outcomes in patients with primary aldosteronism. Jama 295: 2638-2645.

2. Wu VC, Chang HW, Liu KL, Lin YH, Chueh SC, et al. (2009) Primary Aldosteronism: Diagnostic Accuracy of the Losartan and Captopril Tests. Am J Hypertens 22: 821-827.

3. Kuo CC, Wu VC, Huang KH, Wang SM, Chang CC, et al. (2011) Verification and evaluation of aldosteronism demographics in the Taiwan Primary Aldosteronism Investigation Group (TAIPAI Group). J Renin Angiotensin Aldosterone Syst 12: 348-357.

4. Chao CT, Wu VC, Kuo CC, Lin YH, Chang CC, et al. (2013) Diagnosis and management of primary aldosteronism: an updated review. Ann Med 45: 375-383.

5. Wu VC, Yang SY, Lin JW, Cheng BW, Kuo CC, et al. (2011) Kidney impairment in primary aldosteronism. Clin Chim Acta 412: 1319-1325.

6. Yen RF, Wu VC, Liu KL, Cheng MF, Wu YW, et al. (2009) 131I-6beta-iodomethyl-19-norcholesterol SPECT/CT for primary aldosteronism patients with inconclusive adrenal venous sampling and CT results. J Nucl Med 50: 1631-1637.

7. Wu VC, Chao CT, Kuo CC, Lin YH, Chueh SC, et al. (2012) Diagnosis and Management of Primary Aldosteronism. Acta Nephrologica 26: 111-120.
